# Gain roll-off in cadmium selenide colloidal quantum wells under intense optical excitation

**DOI:** 10.1038/s41598-022-11882-6

**Published:** 2022-05-16

**Authors:** Benjamin T. Diroll, Alexandra Brumberg, Richard D. Schaller

**Affiliations:** 1 Center for Nanoscale Materials, Argonne National Laboratory, 9700 S. Cass Avenue, Lemont, IL 60439 USA; 2 Department of Chemistry, Northwestern University, 2145 Sheridan Road, Evanston, IL 60208 USA

**Keywords:** Condensed-matter physics, Materials for optics, Nanoscale materials

## Abstract

Colloidal quantum wells, or nanoplatelets, show among the lowest thresholds for amplified spontaneous emission and lasing among solution-cast materials and among the highest modal gains of any known materials. Using solution measurements of colloidal quantum wells, this work shows that under photoexcitation, optical gain increases with pump fluence before rolling off due to broad photoinduced absorption at energies lower than the band gap. Despite the common occurrence of gain induced by an electron–hole plasma found in bulk materials and epitaxial quantum wells, under no measurement conditions was the excitonic absorption of the colloidal quantum wells extinguished and gain arising from a plasma observed. Instead, like gain, excitonic absorption reaches a minimum intensity near a photoinduced carrier sheet density of 2 × 10^13^ cm^−2^ above which the absorption peak begins to recover. To understand the origins of these saturation and reversal effects, measurements were performed with different excitation energies, which deposit differing amounts of excess energy above the band gap. Across many samples, it was consistently observed that less energetic excitation results in stronger excitonic bleaching and gain for a given carrier density. Transient and static optical measurements at elevated temperatures, as well as transient X-ray diffraction of the samples, suggest that the origin of gain saturation and reversal is a heating and disordering of the colloidal quantum wells which produces sub-gap photoinduced absorption.

Colloidal quantum wells (CQWs), frequently called nanoplatelets, are promising materials for solution-based optical gain media. CdSe CQWs exhibit low thresholds for amplified spontaneous emission and lasing spanning visible wavelengths^1–6^ and the largest modal gain of any nanomaterials^7^, enabling even reported continuous wave operation^3,8^. Driven by large exciton binding energies, optical gain and lasing action in CdSe CQWs is reported to occur due to a biexcitonic state, similar to other colloidal quantum dots^3,9^. This is distinct from most epitaxial quantum wells in which gain, particularly near ambient temperatures, is most commonly observed from free carrier plasmas^10–13^. This work explores the possibility of generating a carrier plasma in CdSe CQWs under intense optical excitation via a Mott transition, in which the density of excitation destabilizes excitons, yielding a carrier plasma. Mott transitions from biexcitonic gain to plasma gain have been observed in related epitaxial quantum wells^14,15^ and nanowires^16^. Previous works have speculated that gain in CQWs may saturate due to a phase space filling with excitons or a Mott transition at high excitation intensities (> 1 mJ∙cm^−2^)^7.^ One literature report indicates the coexistence of both excitonic absorption and electron–hole plasma^17^.

By examining the extinction of CQWs at high fluences in several samples with different thicknesses, however, no plasma is observed. Rather, similar to quantum dots^18^, under increasingly intense optical excitation, biexcitonic gain saturates, diminishes, and even reverts to loss due to the formation of a broadband photoinduced absorption at energies below the band gap. Under the same photoexcitation conditions, the excitonic absorption bleaches up to electron–hole sheet densities of *c.* 2 × 10^13^ cm^−2^ above which more intense excitation results in apparent *increasing* excitonic absorption. A similar phenomenon is observed in all measured samples, including core/shell CQWs. In epitaxial quantum wells, gain saturation may be related to phase space filling of excitons^19^ or formation of electron–hole plasmas^20^. In this case, however, several lines of evidence indicate a thermal origin of the saturation and reversal of gain at high excitation intensities, including experiments using different photon energies of the excitation, static thermal difference measurements, and transient X-ray diffraction.

## Results and discussion

### Light amplification in colloidal quantum wells

**Figure 1:**
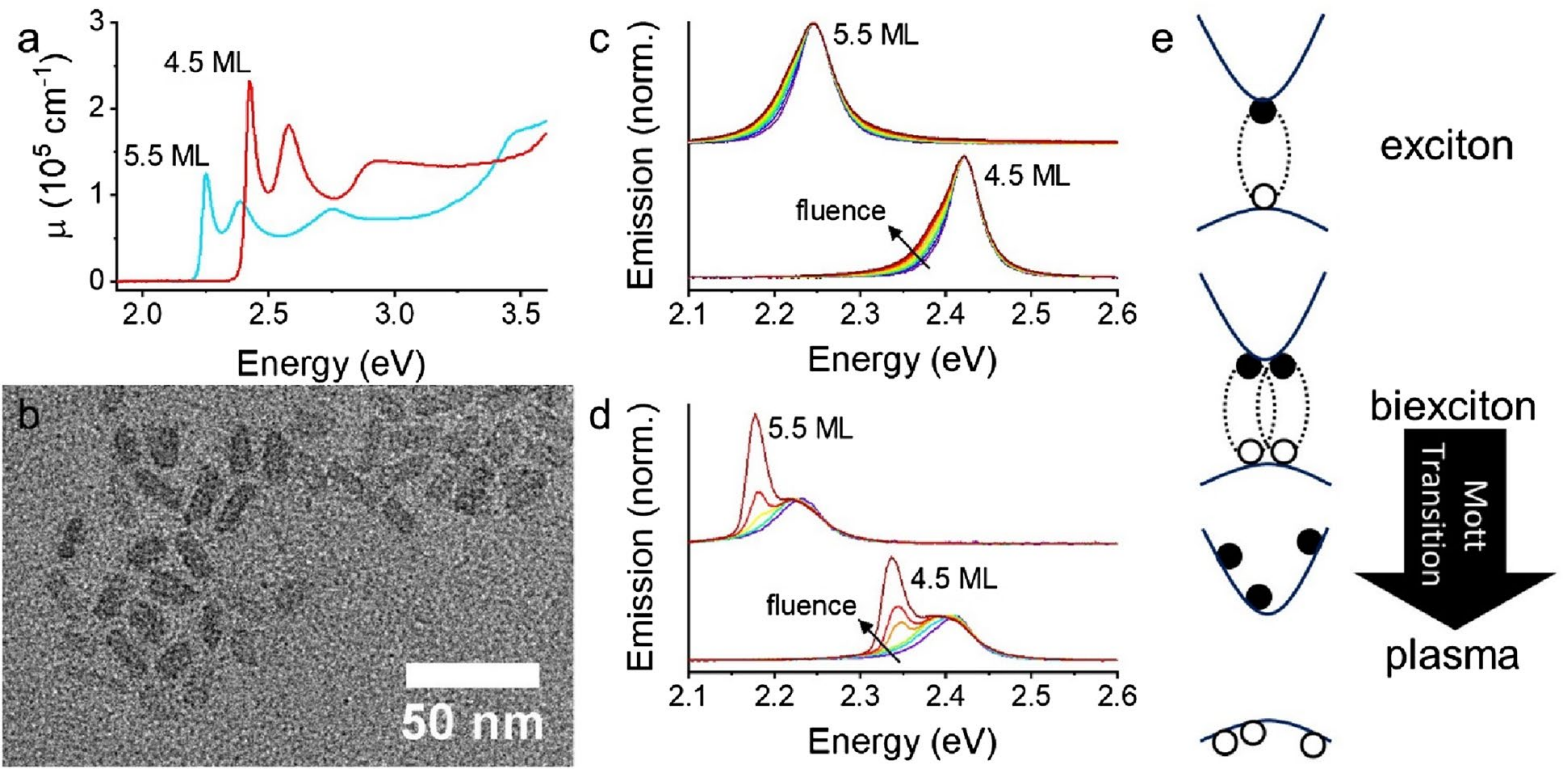
(**a**) Linear absorption (cm^−1^) of 4.5 and 5.5 monolayer (ML) CdSe colloidal quantum wells (CQWs) based upon literature data. (**b**) Typical TEM image of 4.5 ML CdSe CQW sample. (**c**) Normalized, fluence-dependent emission of CQW solutions. (**d**) Normalized, fluence-dependent emission of CQW films illuminated with a 400 μm diameter spot. Normalization is to the excitonic emission peak. (**e**) Cartoon of single exciton, biexciton, and transition to unbound carrier plasma.

Figure 1 shows typical absorption, transmission electron microscopy, and emission of CdSe CQWs. Figure 1a shows the linear absorption (μ) of 4.5 monolayer (ML, 4 monolayers of Se and 5 monolayers of Cd) and 5.5 ML samples based upon literature reports^21^. The monolayer thickness defines the position of the strong excitonic transitions, which arise due to heavy-hole (HH), light-hole (LH), and split-off bands^22^. Atomic precision in thickness across the sample, despite polydisperse lateral dimensions as shown in Fig. 1b, yields narrow optical transitions in absorption and emission. Figure S1 contains transmission electron microscope images of other samples featured in this work. Fluence-dependent emission of dilute solutions of CdSe CQWs (Fig. 1c) shows a pronounced broadening and increased emission at lower energies attributed to biexcitonic emission^3^. In a dense film as in Fig. 1, an amplified spontaneous emission (ASE) feature also emerges above threshold fluences at energies ~ 50 meV lower than the photoluminescence peak.

The origin of ASE in CdSe CQWs has been intensively studied. Underpinning the reported physics of CQW lasing is that they have exciton binding energies far greater than thermal energy at 300 K. Reported exciton binding energies for 4.5 ML and 5.5 ML CQWs are 160–200 meV^23–25^, which is sufficiently large that excitons are anticipated to dominate the physics of these samples up to the melting point^26^. As a consequence, gain and lasing in CQWs is observed from biexcitonic species (at times termed “excitonic molecules”), as shown in the cartoon in Fig. 1e^1,3,27^. The exact number of excitons per particle corresponding to the transparency condition ($\mathrm{A}+\Delta \mathrm{A}=0$) and enabling gain depends on the lateral area of the CQWs, but corresponds to an electron–hole density of *c.* 2.5 × 10^12^ cm^−2^ in 4.5 ML CQWs^27^. All previous reports of ASE or lasing have been consistent with this biexcitonic mechanism. This is distinct from epitaxial quantum wells in which biexciton lasing may be observed at low temperatures in some samples, but plasma based lasing is ordinarily found at higher temperatures for which thermal energy is greater than the exciton binding energy^11,28,29^. In principle, gain from a plasma can also be observed above the Mott transition at which electron–hole densities reach such a level that the available space per exciton is comparable to the Bohr radius^20^. Such high excitation densities destabilize excitons and result in an electron–hole plasma. Mott transitions have been observed in bulk and quantum forms of GaAs^16,30,31^, ZnO^32,33^, CdS^32^, and InGaAs^14^, and, despite very large exciton binding energies, in transition metal dichalcogenides^34^. The formation of an electron–hole plasma extinguishes excitonic absorption and is accompanied by a blue-shift of photoluminescence intensity and absorption associated with the continuous density of states of free carriers^14,19,30^. However, there is no unambiguous evidence from photoemission experiments or gain spectroscopy that full Mott transitions occur—and, if so, at what densities—in CQWs. Only one report, based upon transient absorption spectroscopy, indicates the formation of a plasma which nonetheless coexists with biexcitonic gain^17^.

### Gain spectroscopy of colloidal quantum wells

**Figure 2:**
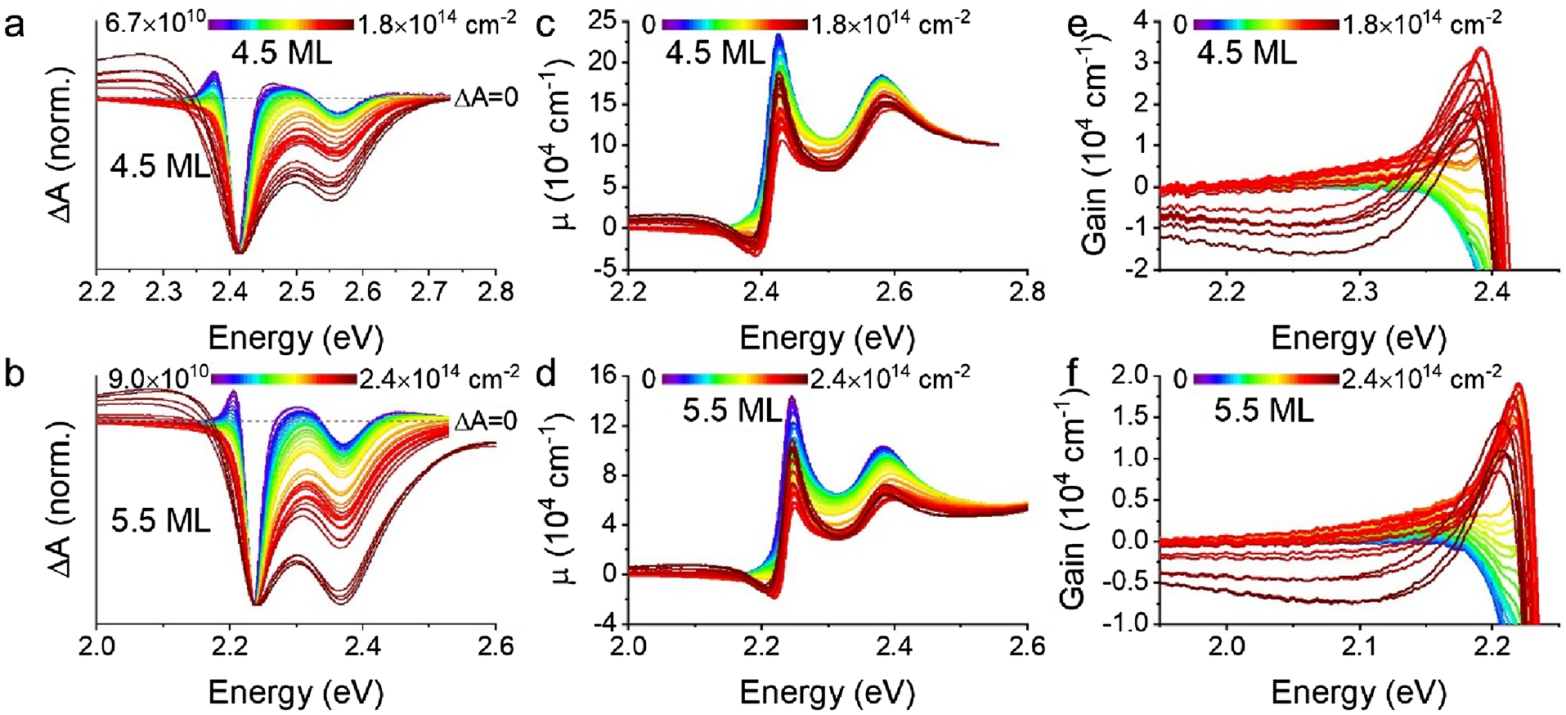
(**a**,**b**) Normalized transient absorption spectra (ΔA) of representative (**a**) 4.5 and (**b**) 5.5 ML CQW samples as a function of photogenerated sheet density. Each spectrum is collected at a 3 ps pump-probe delay for many powers of 3.5 eV pump light. (**c**,**d**) Linear absorption (cm^−1^) calculated for many photogenerated sheet densities of the same (**c**) 4.5 and (**d**) 5.5 ML samples. (**e**,**f**) Zoomed in regions of (**c**) and (**d**), respectively, showing the spectral window of gain.

To examine the formation of an electron–hole plasma in CdSe CQWs, transient absorption was performed on several 4.5 ML and 5.5 ML samples at variable excitation fluence. To ensure, as best as possible, that the results do not reflect photocharging or irreversible sample changes, samples were vigorously stirred during measurements and data presented in this work represents reproducible transient spectra of multiple time delay scans. The results of these experiments, with transient spectra collected at 3 ps pump-probe delay, which allows relaxation of photoexcited carriers, are shown in normalized plots in Fig. 2a and b. (Corresponding data collected at 40 ps and 3 ns pump-probe delay may be found in Figs. S2 and S3.) Raw ΔA data is presented in Fig. S4. Several phenomena occur in both fluence-dependent series: (i) initially narrow bleaching features broaden substantially at carrier density up to *c.* 2 × 10^13^ cm^−2^; (ii) the bleaching intensity of the light-hole feature continuously increases relative to the heavy-hole band edge bleach with increasing fluence; and (iii) at electron–hole densities greater than 2 × 10^13^ cm^−2^, a broad photoinduced absorption appears at energies below the excitonic absorption. The photoinduced absorption or gain for the two samples are shown in Fig. 2c and d, respectively, with an expansion of the spectral region showing gain in Fig. 2e and f. As shown in Fig. 2c and d (and Fig. S4), the excitonic bleach feature of the CQWs first decreases, due to bleaching, but this effect saturates and then reverses with increasing fluence. A related effect is apparent in the gain. Earlier reports of the gain spectrum of CdSe CQWs show very similar gain spectra, including saturation, for electron–hole densities of *c.* 1 × 10^13^ cm^−2^ and lower^1,17,27,35,36^, which corresponds roughly, depending on the CQW size and excitation photon energy, with fluences > 500 μJ∙cm^−2^ or exciton numbers > 30 per CQW. Most earlier literature does not report results for higher excitation densities, but in one report from Tomar et al.^17^, a second band of gain is observed at 2.45 eV for 4.5 ML samples. This was not observed in the present study for any of several samples, including (as shown in Fig. S5) core/shells: at the highest carrier densities, the photoinduced absorption results in substantially diminished gain bandwidth, similar to observed photoinduced absorptions in CdSe quantum dots^18^, and no second band of gain is observed. The absence of the second gain band suggests that an electron–hole plasma is not achieved and the gain mechanism remains excitonic. This is particularly surprising for core/shell samples, which are anticipated to have much reduced exciton binding energies.

**Figure 3:**
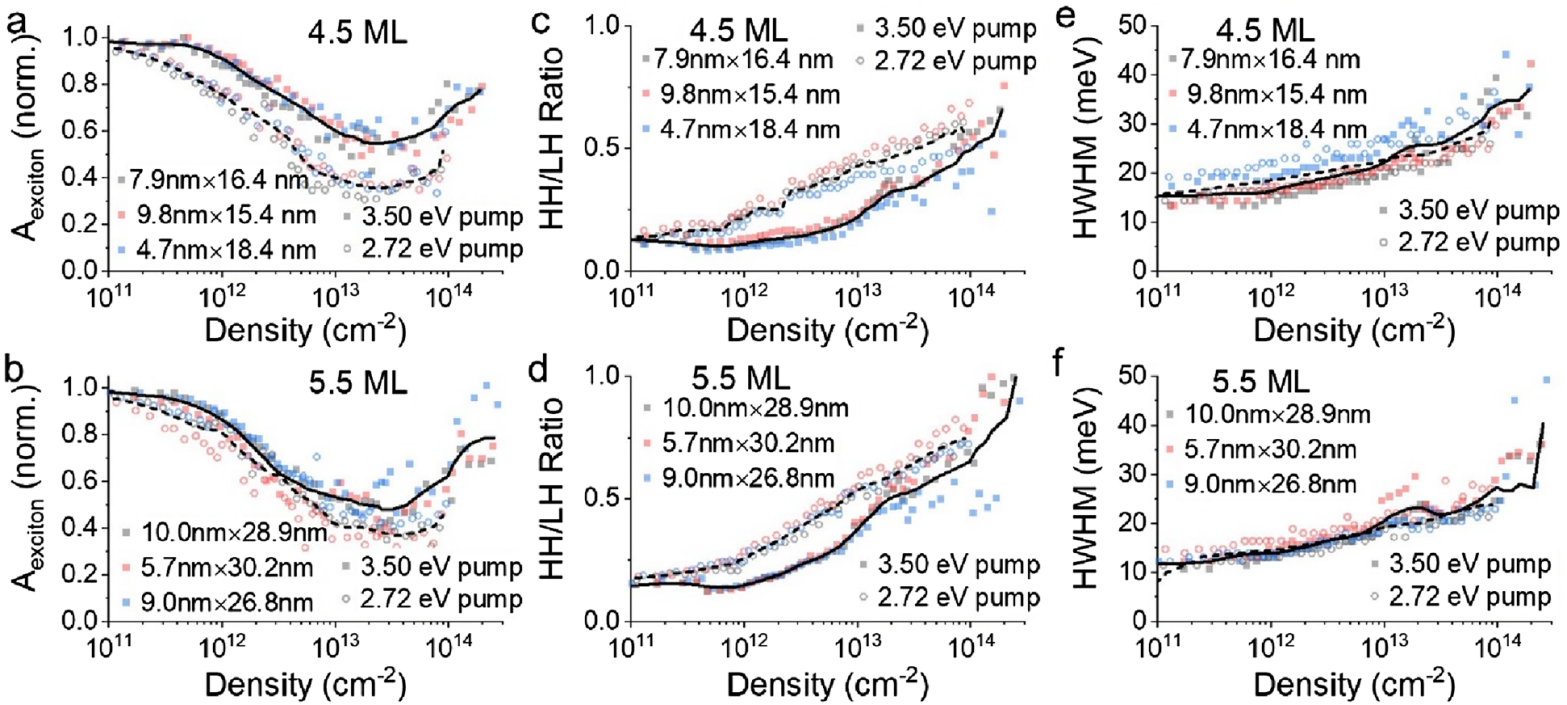
(**a**,**b**) Normalized exciton absorption intensity for three (**a**) 4.5 and (**b**) 5.5 ML CQW samples as a function of photogenerated sheet density. Each data point is extracted from a transient absorption spectrum collected at 3 ps pump-probe delay for many powers of 2.72 eV and 3.50 eV pump light. (**c**,**d**) Ratio of heavy hole and light hole bleach signals as a function of photogenerated sheet densities for the same (**c**) 4.5 and (**d**) 5.5 ML samples. (**e**,**f**) Half-width at half-maximum (HWHM) of the HH bleach feature from the transient absorption spectrum (ΔA) plotted against photogenerated sheet densities of the same (**e**) 4.5 and (**f**) 5.5 ML samples. In all cases, data for measurements with 3.50 eV pump are shown in solid symbols and data for 2.72 eV pump measurements are shown in open symbols. A solid line represents a smoothed average of 3.50 eV pump experiments and a dashed line corresponds to the smoothed average of 2.72 eV pump.

**Figure 4:**
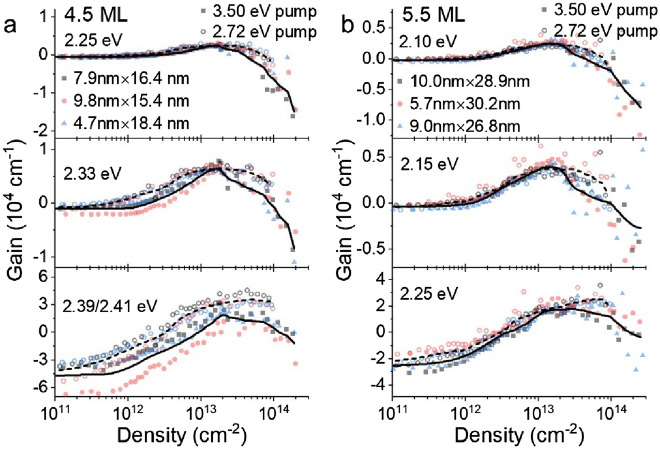
(**a**,**b**) Line-cuts of gain or loss at 3 ps pump-probe delay for representative energy values as a function of photogenerated sheet density for (**a**) 4.5 ML and (**b**) 5.5 ML CQW samples. In all cases, data for measurements with 3.50 eV pump are shown in solid symbols and data for 2.72 eV measurements are shown in open symbols. A solid line represents a smoothed average of 3.50 eV pump experiments and a dashed line corresponds to the smoothed average of 2.72 eV pump.

The trends apparent in Fig. 2 are quantified in Figs. 3 and 4 across three samples each of 4.5 ML and 5.5 ML thicknesses using two excitation photon energies, 3.50 eV and 2.72 eV (see Fig. S6). Here, data are presented as a function of the electron–hole sheet density of the samples, rather than fluence, which does not account for energy differences of the pump excitations, or exciton number, which does not account for differences in the CQW physical dimensions. These alternative representations may be found in Figs. S7 and S8. Individual points show the data for different CQW samples and the solid and dashed lines show the smoothed averaged data of all the samples with either 3.50 eV (solid) or 2.72 eV (dashed) pump photon energy. Figure 3a and b show the normalized intensity of the first excitonic absorption associated with the heavy hole of 4.5 ML and 5.5 ML CQWs, respectively, as a function of the electron–hole density of the samples. Bleaching of the exciton was consistently greater under photoexcitation with 2.72 eV photons, compared to 3.50 eV photoexcitation, but in both cases, the bleaching saturates and reverses for electron–hole excitation densities greater than 2 × 10^13^ cm^−2^. It is noted that a related phenomenon has been observed in core/shell CQW systems in saturable absorption experiments, attributed to potential exciton-exciton interactions or enhanced upconversion of higher-energy LH excitons from HH excitons^37^. This second explanation is consistent with data in Fig. 3c and d, showing larger LH to HH ratios.

The ratio of LH to HH bleaching intensity is a function of carrier density via state filling and temperature (see below). As shown in Fig. 3c and d, the ratio of the LH bleaching intensity is consistently larger, for similar initial carrier density, for the 2.72 eV pump than the 3.50 eV pump. The stronger intensity of excitonic bleaching of *both* HH and LH transitions under 2.72 eV photon energy excitation may be explained by higher effective quantum yield for bleaching with less energetic photons^38^, although reports of the energy-dependent quantum yield are contested^39–42^. A second effect accompanying more intense photoexcitation is captured in Fig. 3e and f, showing the half-width at half-maximum of the transient absorption heavy hole bleach features. The broadening of the transient absorption bleaching features at high electron–hole densities, which appears largely insensitive to the photoexcitation energy, reflects both multiple exciton physics (*e.g.* the formation of biexcitonic species and concomitant stimulated emission) and increases in lattice temperature, as demonstrated in temperature-dependent spectroscopy below.

The observable gain in CQWs under the same photoexcitation conditions is analyzed at a few representative energies for the 4.5 ML and 5.5 ML samples in Fig. 4. Similar to earlier work^27^, peak gain values, achieved at the highest energies monitored in Fig. 4, reach values of 20,000–30,000 cm^−1^, which is at least qualitatively consistent with the large gain coefficients observed in variable stripe measurements^7^. In all observed cases, similar to the persistent excitonic absorption, a blueshift and broadening of gain associated with a Mott transition is not observed. Instead, at excitation intensities greater than 2 × 10^13^ cm^−2^, modal gain saturates and reverses at all measured wavelengths and is not observed at all for energies larger than the HH excitonic transition. Gain is consistently greater and saturates at slightly higher electron–hole densities using 2.72 eV photoexcitation as compared to 3.50 eV pumping. This effect is stronger in the case of 4.5 ML CQWs, for which the relative difference in excess energy of the pump excitation above the band gap is much larger.

The absence of the spectral signatures of a Mott transition in CQWs under intense excitation is surprising. CdSe colloidal quantum dots show substantial quenching of the first (1S) excitonic absorption^18,43,44^ as do related transition metal dichalcogenides^34^. At electron–hole densities greater than 1 × 10^13^ cm^−2^, the effective radius of carriers is less than one-half of the bulk CdSe Bohr radius (5.6 nm)^45^. One explanation is that the in-plane exciton size in CdSe CQWs is much smaller than the Bohr radius of bulk CdSe, comparable to the CQW thickness^23,46^, or less than one-fifth of the Bohr radius in these samples. Nonetheless, at still higher excitation intensity, a Mott transition remains possible and at the highest excitation intensities used in this work, the effective radius available per exciton is < 1 nm. The persistent strength of excitonic absorption, even for such high carrier densities, and the absence of plasma formation, provide evidence consistent with theoretical predictions of a degenerate quantum exciton gas^47^. Instead of the formation of an electron–hole plasma, photoinduced absorption results in gain reversal at all energies and a narrowing of gain bandwidth. At lower energies such as 2.25 eV for 4.5 ML CQWs or 2.10 eV for 5.5 ML CQWs, the photoinduced absorption yields particularly large losses greater than 10,000 cm^−1^.

It should also be noted that all of the trends apparent at 3 ps pump-probe delay may also be observed at longer time delays. Analogous patterns of excitonic bleaching, broadening, and gain are observed in data collected at 40 ps pump-probe delay, which is shown in Figs. S9 and S10. The pattern of persistently stronger excitonic bleaching and gain in samples pumped with 2.72 eV pump photon energy compared to those with 3.50 eV pump photon energy is preserved at longer pump-probe delays, with differences in excitonic absorption becoming even larger. However, at longer pump-probe delays, the magnitude of most features is weaker and the gain bandwidth smaller as excitonic recombination is rapid once population inversion is achieved.

### Thermal response under intense photoexcitation

Broad, “parasitic” photoinduced absorption was previously observed in CdSe colloidal quantum dot samples^18^. In that work, Malko et al*.* showed that cross section of the photoinduced loss was fixed for all quantum dot sizes, completely suppressing gain in small quantum dots, but not in large quantum dots. Based upon sensitivity of the gain to solvent environment (or solid versus solution conditions), the photoinduced absorption which parasitized optical gain was attributed to extrinsic electronic effects on the quantum dots, such as interfacial trap sites, and not to thermal effects from quantum dot heating. Arguing against a thermal origin of gain reversal in CdSe quantum dots, Malko et al*.* reported no red-shifts of the photoluminescence at high intensities which would be associated with the Varshni-like behavior of the CdSe band gap^18^.

Although the phenomenon observed optically appears to be quite similar in CdSe CQWs and quantum dots, the details are distinct in many respects. In the case of CQWs, several lines of evidence implicate a thermal origin to the reduction of gain at high excitation intensities. A simple calculation based upon the heat capacity of bulk CdSe, assuming no heat dissipation into the environment, and using excitation densities of the experiments presented here indicates that the temperature of the CdSe lattice can increase by 100 K or more for electron–hole densities greater than 1 × 10^14^ cm^−2^, with more heating anticipated for a larger excess photon energy of the pump. (See Supporting Information Fig. S11.) For reference, pulsed excitation fluences 3–4 times greater than those used in this work (13–17 mJ∙cm^−2^) are reported to reversibly melt bulk CdSe^48^, which has a substantially higher melting point^26,49^ and comparable intensities to those used here (as discussed below) were found by transient X-ray diffraction of nanocrystals to yield disordering^50^.

The rate of heat dissipation to the environment is therefore critical. Heat outflow from CQWs to methylcyclohexane, which is used for the gain spectroscopy experiments, occurs, at least for small temperature differentials, on a time-scale of *c.* 160 ps for a 4.5 ML CdSe CQW sample^51^ and *c.* 240 ps (Fig. S12) for a 5.5 ML CdSe CQW sample used in this work. At large temperature differentials, such as those in transient X-ray diffraction, heat loss to a solution environment is also on time-scale of hundreds of picoseconds^52^. Dissipation of heat in the solid state is even slower^51,53,54^. Buildup of gain or ASE occurs with intraband relaxation in ~ 1 ps, based upon time-resolved studies of gain in Figs. S13 and S14 and literature data^2,17,27^. Because the time-scale of heat dissipation is much slower than the buildup of gain, lattice heating of the CQW occurs simultaneously with gain and ASE. At the same time, photoinduced heating of the CQWs may have a relatively small influence on time-integrated emission occurring over several nanoseconds, particularly in solutions. The data presented for 2.72 eV and 3.50 eV photon pump energy are at least indicative of the influence of heating arising from the larger excess energy of the 3.50 eV pump. The gain and excitonic bleaching with 2.72 eV photon energy are stronger and do not reverse as substantially for a given electron–hole density, compared to the 3.50 eV pump.

**Figure 5:**
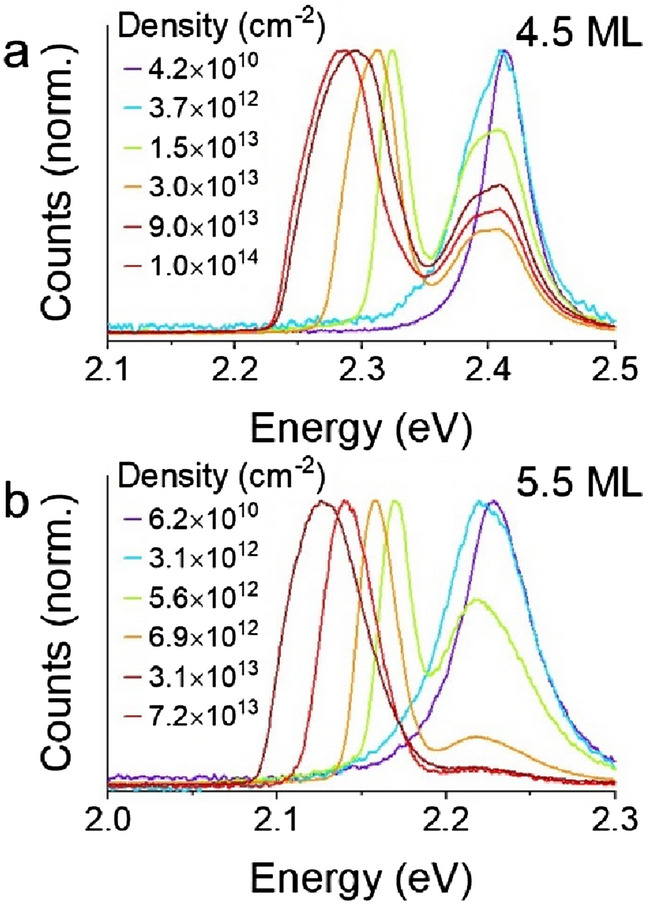
(**a**,**b**) High-fluence photoemission from a circular spot of (**a**) 9.8 nm × 15.4 nm 4.5 ML and (**b**) 9.0 nm × 26.8 nm 5.5 ML CdSe CQWs.

Distinct from earlier reports on quantum dots, the photoluminescence and ASE band of CQWs red-shift appreciably at electron–hole densities greater than 2 × 10^13^ cm^−2^^7,17,27^. High fluence photoemission measurements far above the gain threshold were performed in a front face reflection geometry on semitransparent thin films of CQWs using a small excitation spot to suppress the intensity of ASE and avoid inner filtering effects on the emission. Figure 5 shows the results of high-intensity photoexcitation of CdSe CQW films, in which the band of ASE red-shifts to lower energy with progressively higher fluence. From Figs. 2 and 4, this red-shift is not well-explained by a red-shift in the gain spectrum. Indeed, in the case of 4.5 ML CQWs, the gain band begins to blue-shift as the available gain bandwidth decreases (Fig. 5a). Also noteworthy, the relative intensity of the ASE band compared to the excitonic and biexcitonic emission saturates at electron–hole densities of > 5 × 10^13^ cm^−2^ for the 5.5 ML sample and for the 4.5 ML sample, the relative intensity of ASE even decreases. This saturation and reduction of ASE intensity is consistent with the reduction of optical gain at higher electron–hole densities observed by gain spectroscopy on solutions.

**Figure 6:**
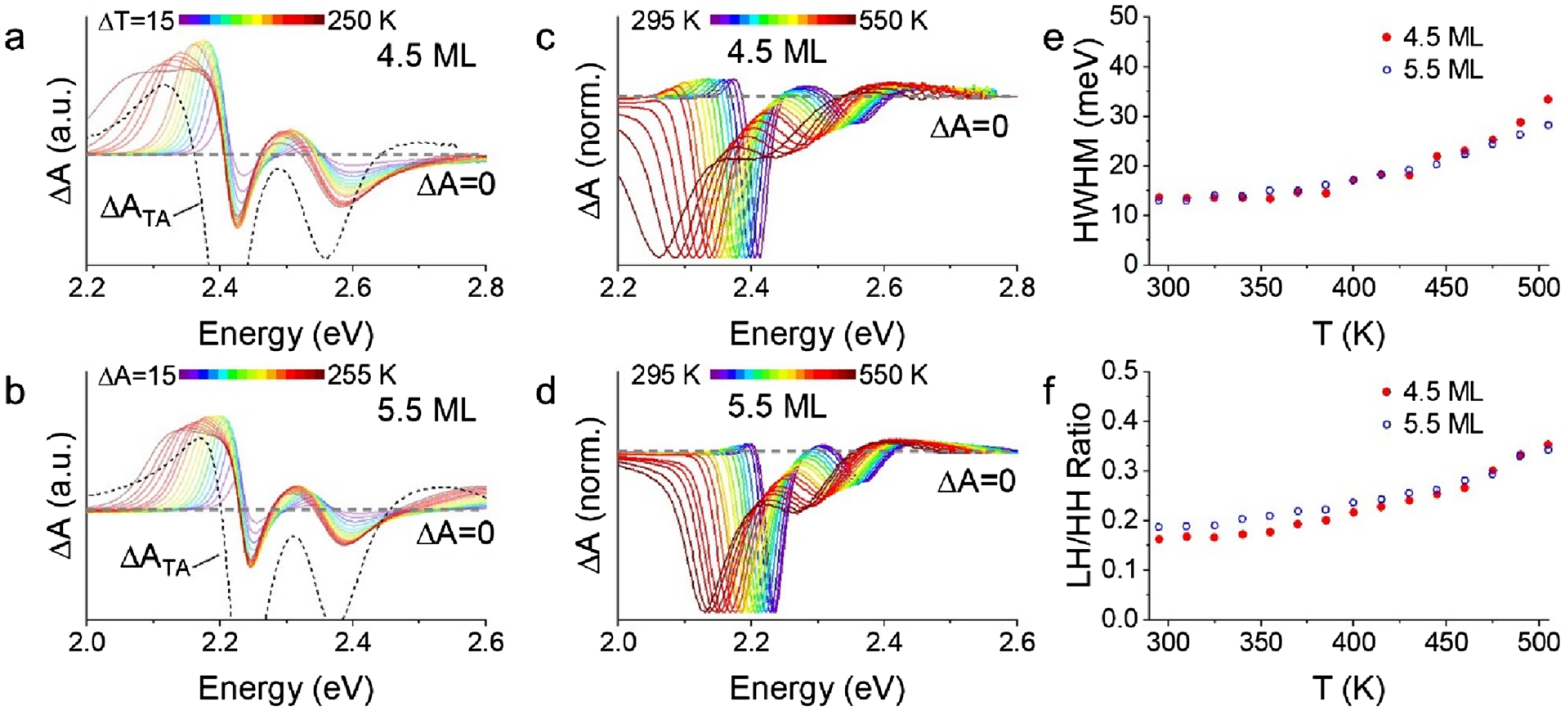
(**a**,**b**) Thermal differential absorption spectra (ΔA = A_T_-A_295 K_) of (**a**) 9.8 nm × 15.4 nm 4.5 ML and (**b**) 9.0 nm × 26.8 nm 5.5 ML CdSe CQWs are shown in colors. The black dashed line shows the ΔA of the same 4.5 or 5.5 ML CdSe CQWs under intense photoexcitation (> 5 × 10^13^ cm^−2^). The plots are scaled for presentation. (**c**,**d**) Transient absorption spectra collected at a pump-probe delay of 3 ps and average excitation density < 1 × 10^12^ cm^−2^ as a function of sample temperature. (**e**) Half-width at half-maximum of transient absorption bleach features as a function of temperature based upon data in figure panels (**c**) and (**d**). (**f**) Ratio of the light hole (LH) and heavy-hole (HH) bleach feature as a function of sample temperature using data in (**c**) and (**d**).

Complementing this, static absorption spectra of the 4.5 ML and 5.5 ML CQW samples were also collected (raw data in Fig. S15) and the thermal difference spectra (A_T_-A_295 K_) are shown in Fig. 6a and b overlapped with a transient absorption spectrum collected at high fluence. As anticipated from the thermochromic behavior of CdSe^26^, increases in the static temperature of the CQWs leads to an increase in the absorption of the film at energies below the ambient band gap, qualitatively resembling the photoinduced absorption feature observed by transient spectroscopy. An important distinction should be noted: although the static absorption data show a typical red-shift with heating, the apparent peak of the exciton in gain spectra in Fig. 2 does not shift substantially. This apparent contradiction is explained, primarily, by the presence of stimulated emission from biexcitons (responsible for gain) which suppress linear absorption μ on the red edge of the lowest excitonic absorption feature. This stimulated emission feature, which produces a negative ΔA signal, directly competes with redshifted absorption that produces a positive ΔA signal apparent in Fig. 6a and b at low energies. Other smaller sources of divergence can include Moss-Burstein filling, which blueshifts the spectrum of the CQWs at high sheet density, and any contributions from the decay of excitation due to Beer’s law, which broadens the temperature profile of the resulting CQWs, resulting in a more gently sloping induced absorption feature in transient absorption measurements than in static thermal difference spectra.

In addition to the changes below band gap, both static absorption spectra and low-fluence (< 1 × 10^12^ cm^−2^) transient absorption spectra (Figs. S15, 6c and d) collected at elevated sample temperatures show increases in the LH:HH ratio and the band-width of the photoinduced bleach which are catalogued in Fig. 6e and f, respectively. In particular, the band-width of the transient bleach feature under low fluence at 500 K reaches 25–30 meV, close to the same values reached for photoexcited samples at room temperature with electron–hole densities greater than 1 × 10^14^ cm^−2^. Such static data may be used to interpret the time-resolved data, implicating lattice heating (in addition to band filling) as an origin of higher LH:HH ratios, due to increased thermal occupation, and broadening and redshifting of the absorption features.

**Figure 7:**
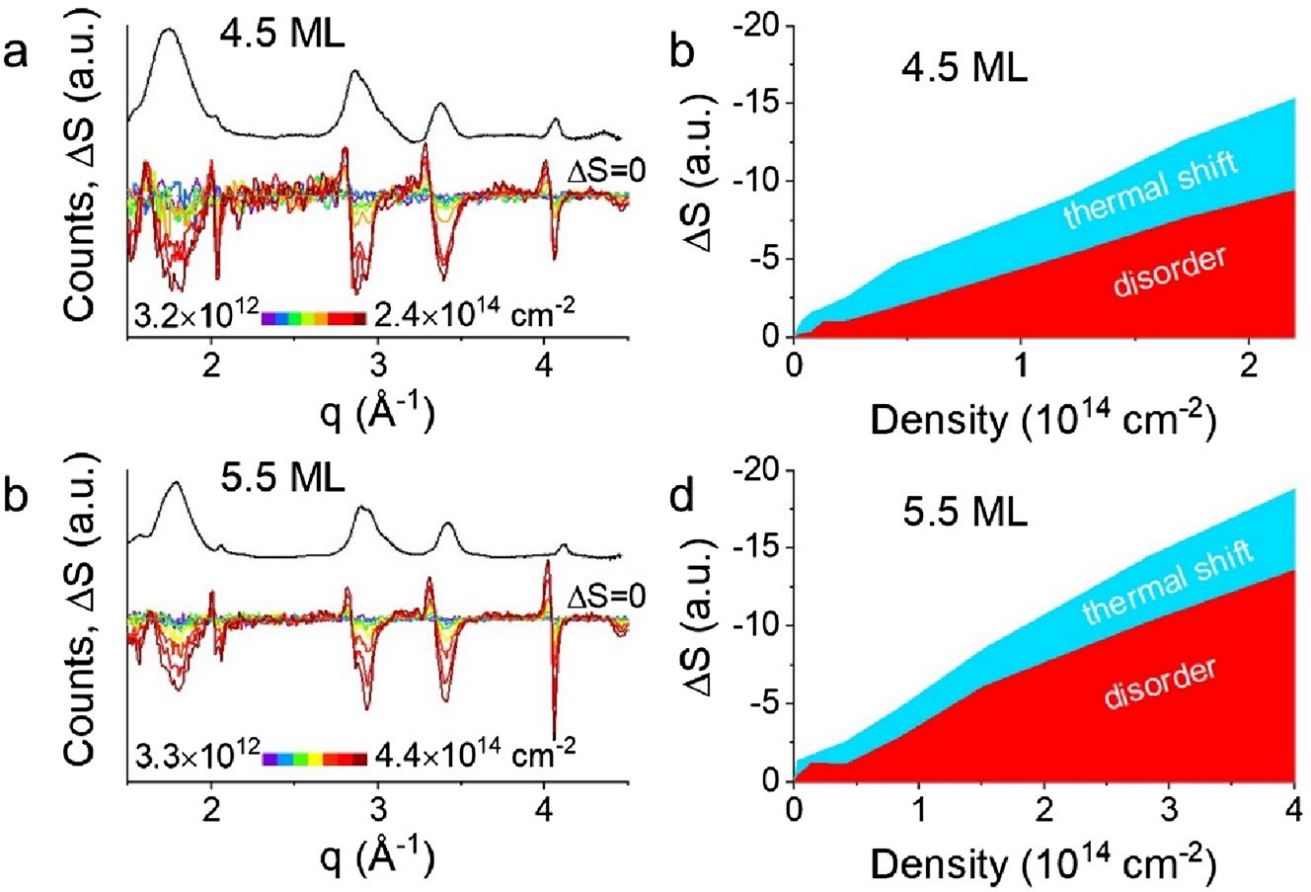
(**a**,**b**) Static X-ray diffraction pattern (black line) and transient X-ray diffraction patterns (ΔS) as a function of power at 40 ps pump-probe delay using 3.1 eV pump photon energy. Data are shown for (**a**) 7.4 nm × 23 nm 4.5 ML and (**b**) 7.6 nm × 41 nm 5.5 ML CdSe CQW samples. (**c**,**d**) Quantification of the magnitude of ΔS signal as a function of excitation density for the same samples. Total ΔS signal is disambiguated into signal arising from thermal disordering and shift of the diffraction angle.

Finally, we highlight that there is strong evidence from dynamic measurements of crystallographic structure that CQWs undergo substantial heating and disordering under photoexcitation^50^. Transient X-ray diffraction patterns of 4.5 ML and 5.5 ML CdSe CQWs are shown in Fig. 7a and b. These data convey the change in X-ray diffraction scattering, ΔS versus *q*, 40 ps after photoexcitation with 3.10 eV photons overlaid on the static, room temperature diffraction pattern of the sample. As detailed elsewhere, the time-resolved ΔS signal can be broken into two contributions from thermal shifts—which results in close to symmetrical derivative-like ΔS contributions—and disorder or phase transitions, which result in changes in the intensity of diffraction peaks^50,52,55,56^. Although previous work has highlighted that disordering occurs preferentially in the short axis of the CQWs^50^, Fig. 7c and d show a simplified integration of ΔS signal attributable to disorder and thermal shift by summing contributions of all available diffraction peaks. The transient X-ray diffraction data shows that at comparable electron–hole excitation densities to the emergence of photoinduced absorption in optical experiments, CQWs show both pronounced heating and disordering of the CQWs. Also, limited temporal dynamics of the transient X-ray diffraction signal (shown in Fig. S16) closely match the dynamics of photoinduced absorption in the same 5.5 ML sample at similar electron–hole densities. As noted above, heating produces a predictable bathochromic shift of the CQW band gap. The optical properties of CQWs in a molten or substantially disordered state have not been measured experimentally, but calculations of the disordered density of states of CdSe nanoparticles also show pronounced reductions of the band gap^52^. Therefore, the transient X-ray diffraction data indicating photoinduced heating of the CQWs lattice is broadly consistent with attributing the observed parasitic photoinduced absorption.

## Conclusions

Collectively, the data presented in this work do not show any indication that the CQWs undergo an electronic transition from an exciton gas to an electron–hole plasma. These data relate lack of a Mott transition in CdSe CQWs under optical excitation, as photoinduced heating at such intensities alters the structure and optoelectronic properties of the CQW. Lattice heating results in saturated gain and, at still higher excitation densities, large optical losses due to photoinduced absorption of hot CQWs. Although resonant excitation at the HH transition is most likely to generate a Mott transition, due to the minimized energy in excess of the band gap, it remains unlikely that without modification of the thermal interfaces of the CQW system that such an optical excitation scheme can generate a Mott transition due to lattice heating arising from Auger processes^57,58^. This does not preclude the possibility of generating a unipolar plasma, which is potentially more promising. These results also emphasize the important role that heat dissipation can play in the performance of nanocrystal-based optoelectronics. Enhancements of the thermal outflow from CQWs to the environment should allow the realization of even higher levels of gain saturation. These results further underline that under high excitation conditions for lasers and bright light emitting diodes, thermal management is a critical element device optimization.

## Supplementary Information

Supplementary Information.
