# Supplementary material for: Gain roll-off in cadmium selenide colloidal quantum wells under intense optical excitation
Source: Sci Rep. 2022 May 16;12:8016. doi: 10.1038/s41598-022-11882-6 (PMC9110332; doi:10.1038/s41598-022-11882-6)
Supplement: Supplementary file 1 — Supplementary Information. [file 41598_2022_11882_MOESM1_ESM.docx]

Supporting Information

Gain Roll-off in Cadmium Selenide Colloidal Quantum Wells under Intense Optical Excitation

Benjamin T. Diroll,^1^* Alexandra Brumberg,^2^ and Richard D. Schaller^1,2^

^1^Center for Nanoscale Materials, Argonne National Laboratory, 9700 S. Cass Avenue, Lemont, Illinois 60439, United States

^2^Department of Chemistry, Northwestern University, 2145 Sheridan Road, Evanston, Illinois 60208, United States

*bdiroll@anl.gov

**Materials and Methods**

***Materials.*** Cadmium acetate hydrate (≥99.99 %, Aldrich), cadmium chloride (99.99%, Aldrich), selenium (99.99 %, powder, ~100 mesh, Aldrich), 1-octadecene (90 %, technical grade, Aldrich), oleic acid (90 %, technical grade, Aldrich), trioctylphosphine (90 % , Aldrich), ammonium sulfide (40-48% in water), cadmium nitrate (97 %, Aldrich), and sodium myristate (>98 %, Aldrich) were sourced from commercial suppliers and used as received. Solvents used were sourced from commercial suppliers and were ACS grade or higher.

***Synthesis.*** The synthesis of CdSe CQWs followed established literature protocols.^1^ Cadmium myristate was synthesized by dissolving 1.5 g sodium myristate in methanol (via sonication) and then adding 600 mg of cadmium nitrate under stirring. The methanolic solution immediately becomes cloudy and is then stirred under air for 4 hours, then the white solid was isolated by centrifugation at 5000 rpm. The white precipitate was re-dispersed in methanol and precipitated two more times, then once using acetone. Cadmium myristate was isolated as a white powder after vacuum drying for 16 hours at 60 °C.

3.5 ML CdSe CQWs were synthesized by mixing 240 mg of cadmium acetate, 150 μL oleic acid, and 15 mL of octadecene. The mixture was held under vacuum for 1 hour at 80 °C, then heated under nitrogen to 180 °C, whereupon a 150 μL solution of 1 M selenium dissolved in trioctylphosphine was rapidly injected (789 mg dissolved in 10 mL of trioctylphosphine). The reaction was held at 180 °C under nitrogen for 10 minutes, then cooled to room temperature and precipitated with isopropanol and re-dispersed in methylcyclohexane.

4.5 ML CQWs were synthesized by combining 12 mg Se powder, 170 mg cadmium myristate, and 15 mL octadecene, which are held under vacuum at room temperature for 1 hour, then heated under nitrogen to 240 °C. At 195 °C, under nitrogen counterflow, 40 mg of finely-ground cadmium acetate was added rapidly. After 5 minutes at 240 °C, the reaction was cooled by removing the heating mantle and 2 mL of oleic acid was injected, followed at ~100 °C with 10 mL of toluene. NPLs were precipitated from the reaction medium by centrifugation at 15000 rpm, then re-dispersed into methylcyclohexane. Smaller CQWs were obtained by arresting the reaction by removing heat at 225 °C; larger by allowing the reaction to run for 10 minutes.

5.5 ML CQWs were synthesized by mixing 170 mg cadmium myristate in 14 mL octadecene, holding under vacuum for 1 hour at room temperature, then heated under nitrogen to 250 °C. At 250 °C, a dispersion of 12 mg selenium in 1 mL octadecene (dispersed with sonication and vigorous mixing) was rapidly injected, followed, after 1 minute, by addition of 90 mg cadmium acetate with nitrogen counterflow. The reaction proceeded for an additional 15 minutes. After removal of the heating mantle, 2 mL of oleic acid was injected, followed at ~100 °C with 10 mL of toluene. Samples were isolated by centrifugation of the reaction mixture at 15000 rpm, with the sample re-dispersed and stored in methylcyclohexane. Different sizes were obtained by arresting the reaction after 10 minutes or 20 minutes instead of 15.

CdSe/ZnS core/shell CQWs. Starting with 4.5 ML CQW cores, shells of ZnS were grown using colloidal atomic layer deposition.^2^ The initial 4.5 ML CdSe cores are diluted into 1 mL of hexanes. A 400 μL amount of N-methylformamide (NMF) is added to the centrifuge tube, forming a polar phase. Then, 20 μL of ammonium sulfide solution is added, and the biphasic system vigorously shaken for 1 min until the upper nonpolar layer is colorless and the NMF layer is yellow. The hexane layer is carefully extracted, the sample is rewashed with clean hexanes by shaking, and hexanes are extracted again, then precipitated with addition of 1:1:2 (v/v) NMF/acetonitrile/toluene. The slightly yellow supernatant is decanted, and the pellet redispersed in 200 μL of NMF. Then the sample is reprecipitated with 1:1:4 (v/v) NMF/acetonitrile/toluene. This completes the formation of the sulfur layer. Zn layers are added by dispersing the sulfide-capped CQWs with 400 μL of 0.15 M zinc acetate dihydrate solution in NMF and vigorously shaking for 1 min. Then, the solution is held for 15 min at 50 °C before precipitating with 1:2 (v/v) NMF/toluene. Subsequently, the pellet is dispersed in 400 μL of clean NMF and transferred to 1 mL of hexanes containing 20 μL of oleylamine and 20 μL of oleic acid by vigorously shaking for 2 min and then allowing the biphasic mixture to stand for 5 min at 50 °C. The yellow hexanes solution is carefully extracted into a new centrifuge tube and precipitated with 1:2 (v/v) hexanes/ethanol. The yellow precipitate is redispersed in 1 mL of clean hexanes. This process was repeated to obtain 4 ML of ZnS. To generate a shell of 4 ML of CdS, 0.15 M cadmium acetate hydrate was used instead of zinc acetate.

***Optical Spectroscopy.*** For transient absorption (TA) measurements of gain at variable fluences, samples were prepared in cleaned 1 mm path length cuvettes with stirbars. The measurement was performed with a 5 MHz Ti:sapphire laser (SpectraPhysics) split into pump and probe beams. The probe beam was directed through an electronically controllable delay stage and focused into a sapphire crystal to generate a supercontinuum white light focused on the samples. The pump beam was directed into an optical parametric amplifier (OPA) and converted to either 2.72 eV or 3.50 eV photon energy, determined by measurement of the pump scatter. Samples were vigorously stirred throughout measurements. To eliminate samples in which high excitation fluence degraded or otherwise irreversibly altered the sample, all measurements of the spectra were taken multiple times to confirm that similar signal levels were maintained on a laboratory time-scale. To minimize sample exposure, the pump laser was shuttered except during measurements. Static optical absorption measurements at room temperature, used for calculation of gain, were taken using a Perkin Elmer Lambda 950 instrument on the same cuvette samples used in TA measurements.

As acquired, the units of transient absorption measurements are in change in absorption (ΔA) and the units of absorption are in optical density. Plots of gain spectra represents A+ ΔA. To generate plots as a function of linear absorption, literature data on the dipole or linear absorption (cm^-1^) at the first excitonic maximum was used to calibrate the datasets. Linear absorption is useful for two reasons: first, it is the common unit by which gain is described (cm-1) and second, it permits comparison between samples which have different absolute absorbance and different molar extinction (due to being larger or smaller). Linear absorption can be interconverted with molar extinction and cross section according to

$$\varepsilon\left( E \right)=\frac{N_{A}\sigma\left( E \right)}{1000\ln10}=\frac{N_{A}V\alpha\left( E \right)}{1000\ln10}$$

Where the variables are molar extinction (ε, typically M^-1^cm^-1^), cross sections (σ, typically cm^-2^), or linear absorption (α or μ, typically cm^-1^), volume (V) and Avogadro’s number (N_A_).^3^

Photoemission measurements were performed using similar laser excitation with the emitted light collected using a fiber, directed through a spectrometer, and measured with a CCD array.

For temperature-dependent static spectra and transient absorption, samples were deposited as thin films on a sapphire plate and loaded into an optical oven, which was evacuated for measurements. Samples equilibrated for 5 minutes at each temperature before measurements. Static absorption spectra were collected using a fiber coupled light source and OceanOptics spectrometer. Transient absorption experiments of the spectrum presented as a function of temperature were performed using 3.10 eV photon energy excitation at low excitation intensity.

Estimation of heat outflow time-scale used previously described literature methods^4^ in which infrared excitation was performed at 3.5 μm pump and a supercontinuum white light generated using 800 nm pump excitation focused into a sapphire plate.

***Microscopy*.** Transmission electron microscopy (TEM) was performed on drop-cast samples on carbon coated copper TEM grids using a JEOL 2100F tool.

***X-Ray Diffraction****.* Transient X-ray diffraction patterns were collected at Beamline 11-ID-D of the Advanced Photon Source at Argonne National Laboratory. CQWs were dispersed in dodecane and continuously recirculated using a peristaltic pump. At the region of data acquisition, CQWs were flowed as a jet and excited in an air-free interaction region purged with nitrogen using the 400 nm, frequency-doubled output of a 1.6 ps Ti:sapphire laser operating at 10 kHz. Powder X-ray diffraction was collected using 11.7 keV, 79 ps X-ray pulses in 24 bunch mode and detected on a time-gated Pilatus 2M detector. For the static X-ray diffraction pattern of 5.5 ML NPLs, a diffraction pattern collected from sector 11 under the condition of no laser excitation was used. For the static X-ray diffraction pattern of 4.5 ML CQWs, the diffraction pattern was collected at sector 5 (DND-CAT) of the Advanced Photon Source at Argonne National Laboratory. The 4.5 ML sample was dispersed in dodecane and placed in a 1.5 mm borosilicate capillary. The X-ray energy was 17 keV. Diffraction was collected on three Pilatus 2M detectors. For both 4.5 and 5.5 ML CQWs, a polynomial baseline was fit to the static diffraction pattern to account for the dodecane background and subtracted out.

**Conversion between cross-section, extinction, and molar absorptivity**

Cross section (σ, in units of cm^2^), linear extinction (μ, in units of cm^-1^), and molar absorptivity (ε, M^-1^∙cm^-1^) are converted using the following equation:^3^

$$\varepsilon=\frac{N_{A}\sigma}{1000 ln 10}=\frac{N_{A}V_{CQW}\mu}{1000 ln 10}$$

Where $N_{A}$ is Avogadro’s number and $V_{CQW}$ is the volume of the CQW.

**Conversion between average number of excitons per particle and sheet density**

The average number of excitons per particle, denoted $\left\langle N \right\rangle$, may be determined using the cross section. For example, $\left\langle N \right\rangle=\sigma n$, where *n* is the number of photons per square centimeter. This may be converted to an electron-hole sheet density by using the lateral dimensions ($A_{CQW}$) of the CQWs as, $2\left\langle N \right\rangle/A_{CQW}$. Sheet density (in cm^-2^) is preferred in this work as a means of describing the electron-hole density because it permits straightforward comparison between samples of different dimensions.

**Supporting Data and Figures.**


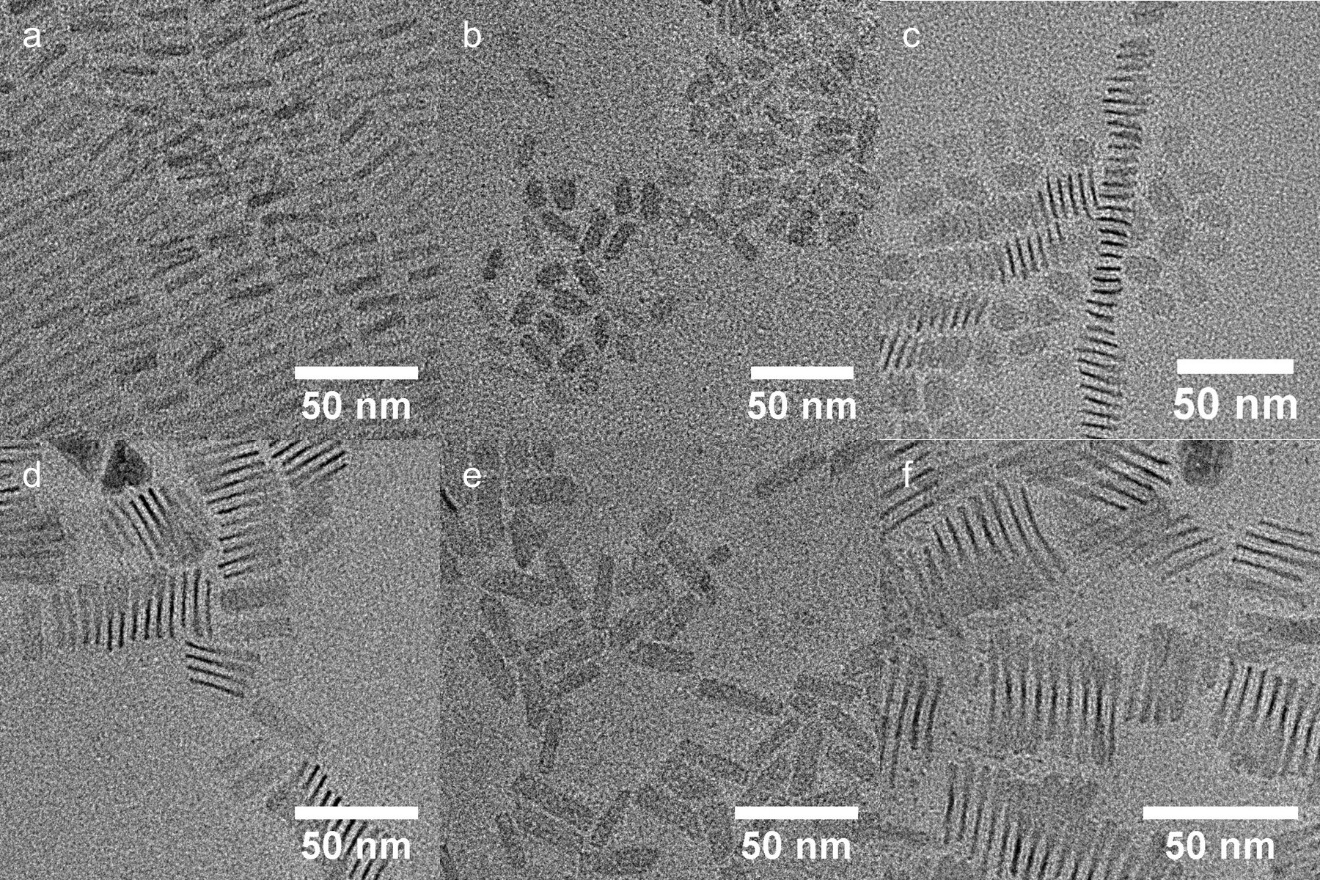


Figure S1. Representative TEM images of (a-c) 4.5 ML CQWs and (d-f) 5.5 ML CQWs.


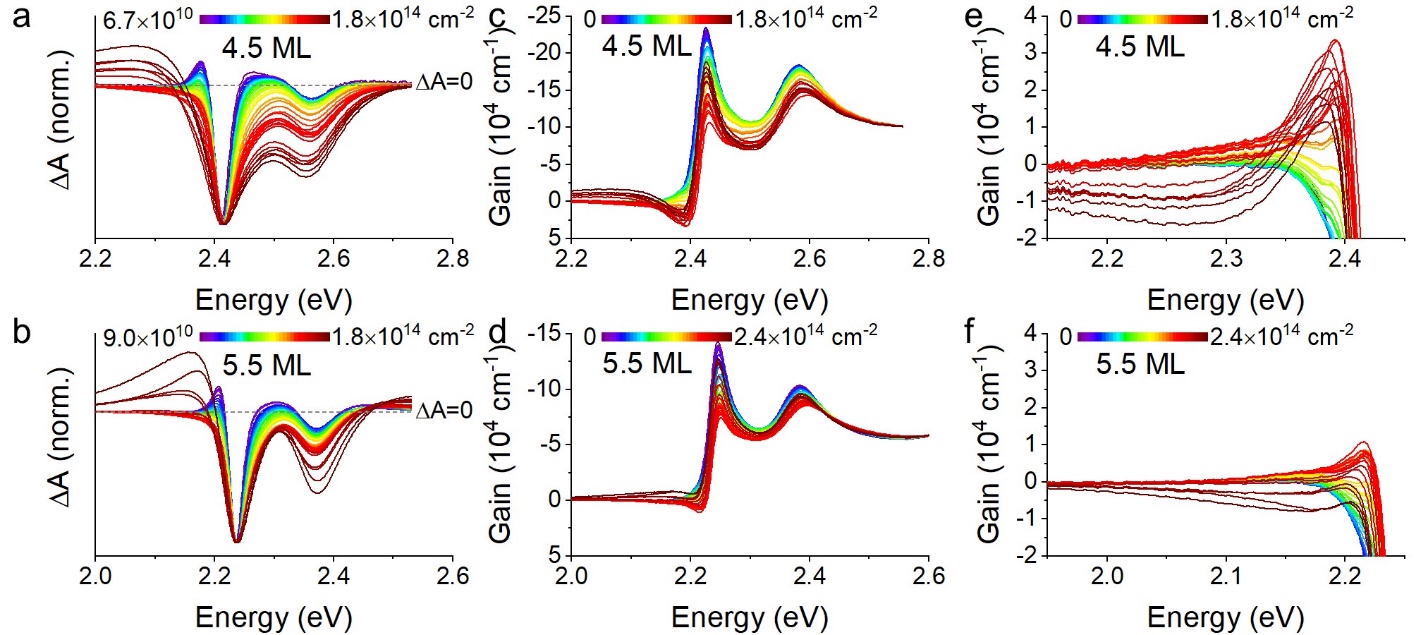


Figure S2. (a, b) Normalized transient absorption spectra (ΔA) of a representative (a) 4.5 and (b) 5.5 ML CQW samples as a function of photogenerated sheet density. Each spectrum is collected at a 40 ps pump-probe delay for many powers of 3.5 eV pump light. (c, d) Linear absorption (cm^-1^) calculated for many photogenerated sheet densities of the same (c) 4.5 and (d) 5.5 ML samples. (e, f) Zoomed in regions of (c) and (d), respectively, showing the spectral window of gain.


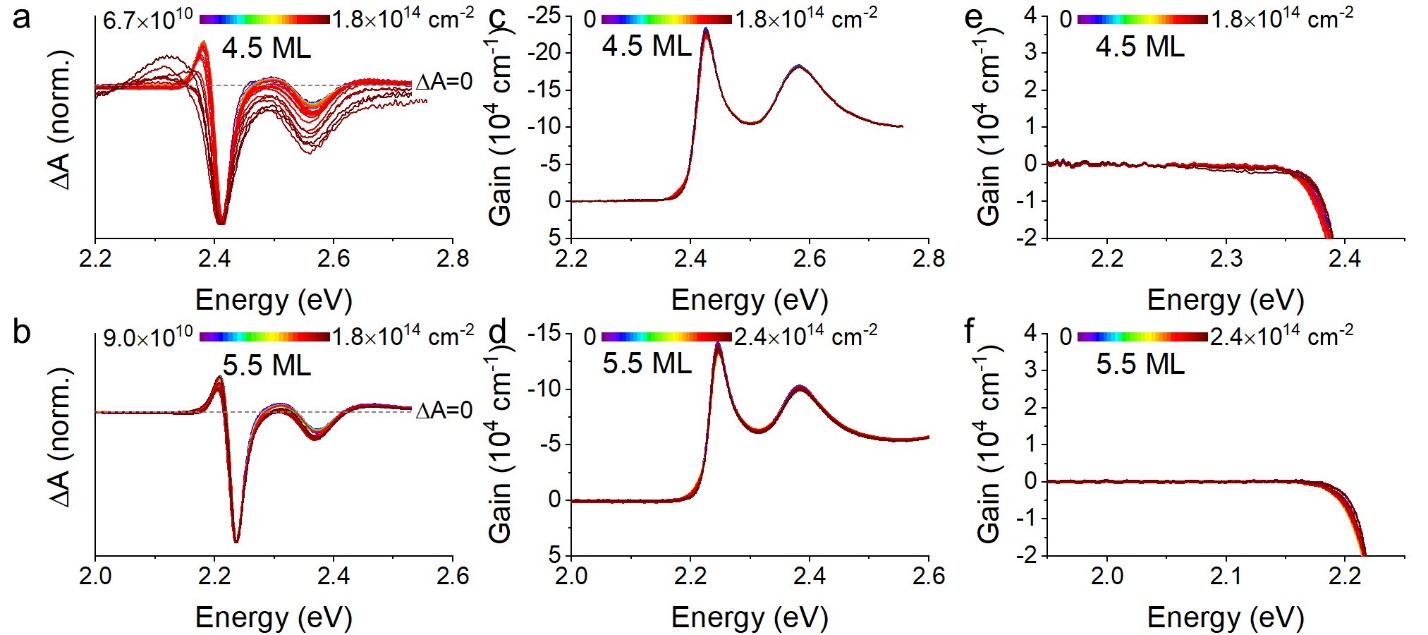


Figure S3. (a, b) Normalized transient absorption spectra (ΔA) of a representative (a) 4.5 and (b) 5.5 ML CQW samples as a function of photogenerated sheet density. Each spectrum is collected at a 3 ns pump-probe delay for many powers of 3.5 eV pump light. (c, d) Linear absorption (cm^-1^) calculated for many photogenerated sheet densities of the same (c) 4.5 and (d) 5.5 ML samples. (e, f) Zoomed in regions of (c) and (d), respectively, showing the spectral window of gain.


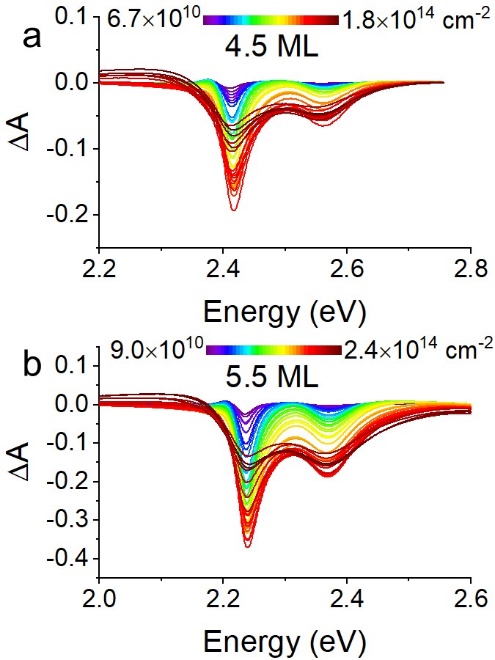


Figure S4. Raw transient absorption data as a function of photogenerated excitation density using a 3.50 eV pump for (a) 4.5 ML CQWs and (b) 5.5 ML CQWs. Normalized data is presented in Figure 2 of the main text.


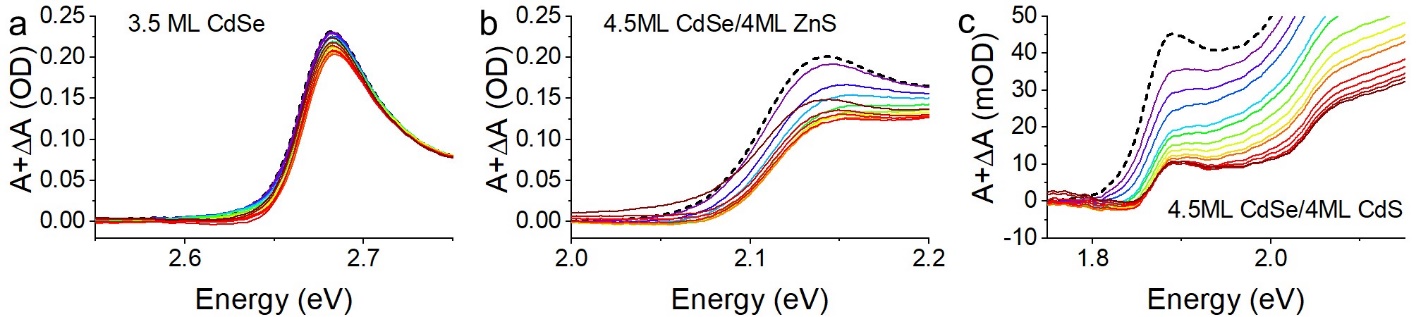


Figure S5. (a-c) Gain spectra of (a) 3.5 ML CdSe, (b) 4.5 ML CdSe/4 ML ZnS, and (c) 4.5 ML CdSe/4 ML CdS CQW samples as a function of 3.50 eV photon excitation intensity, shown increasing from violet to red. A black dashed line indicates the static absorption spectra of the samples.


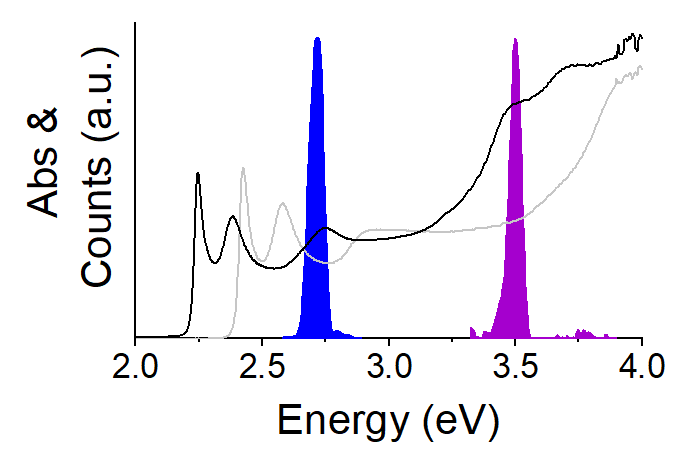


Figure S6. Absorption spectra of representative 4.5 ML and 5.5 ML CdSe CQWs overlaid with the pump scattering spectrum of 2.72 eV and 3.50 eV pump excitations. The energy of the pump photon energies are given in the text based upon the experimentally observed peak of the scattered light spectrum.


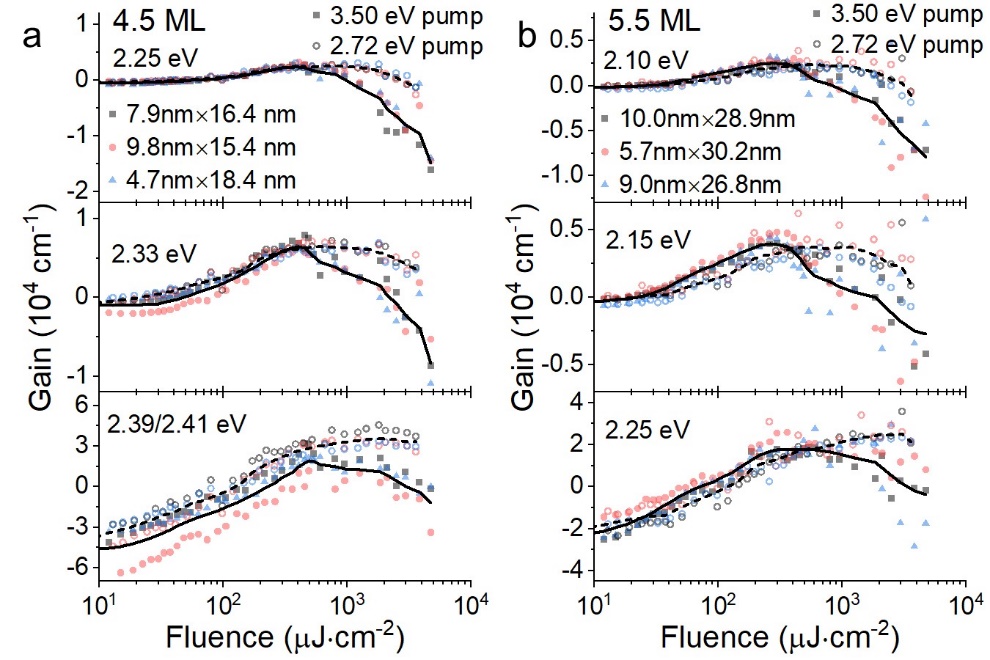


Figure S7. (a, b) Line-cuts of gain or loss at 3 ps pump-probe delay for representative energy values as a function of pump fluence for (a) 4.5 ML and (b) 5.5 ML CQW samples. In all cases, data for measurements with 3.5 eV pump are shown in solid symbols and data for 2.72 eV pump measurements are shown in open symbols. A solid line in (a) and (b) represents a smoothed average of 3.5 eV pump experiments and a dashed line corresponds to the smoothed average of 2.72 eV pump.


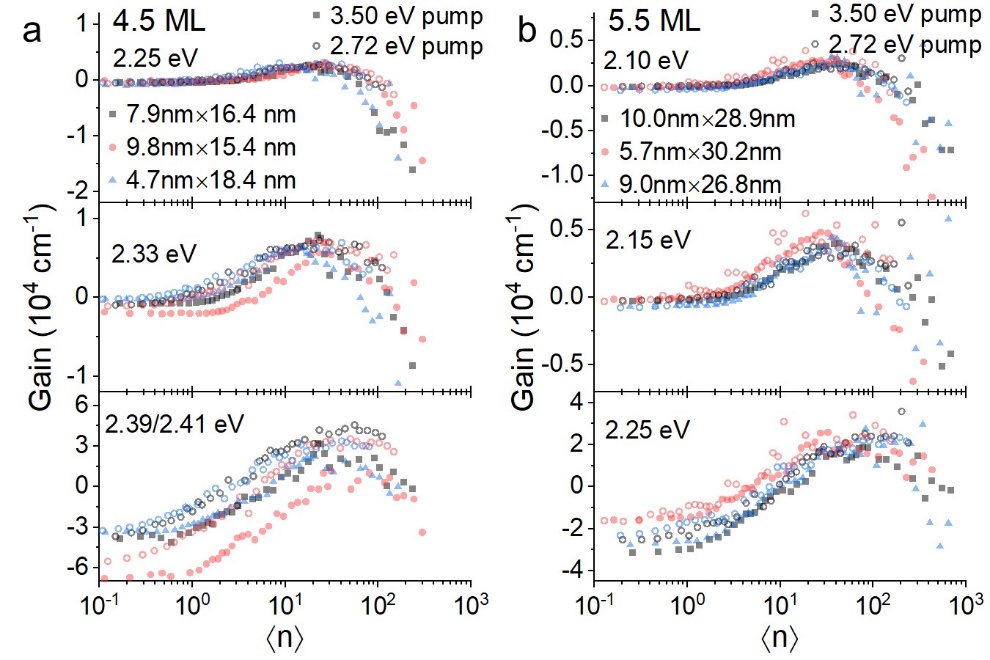


Figure S8. (a, b) Line-cuts of gain or loss at 3 ps pump-probe delay for representative energy values as a function of excitons per CQW for (a) 4.5 ML and (b) 5.5 ML CQW samples. In all cases, data for measurements with 3.5 eV pump are shown in solid symbols and data for 2.72 eV pump measurements are shown in open symbols.


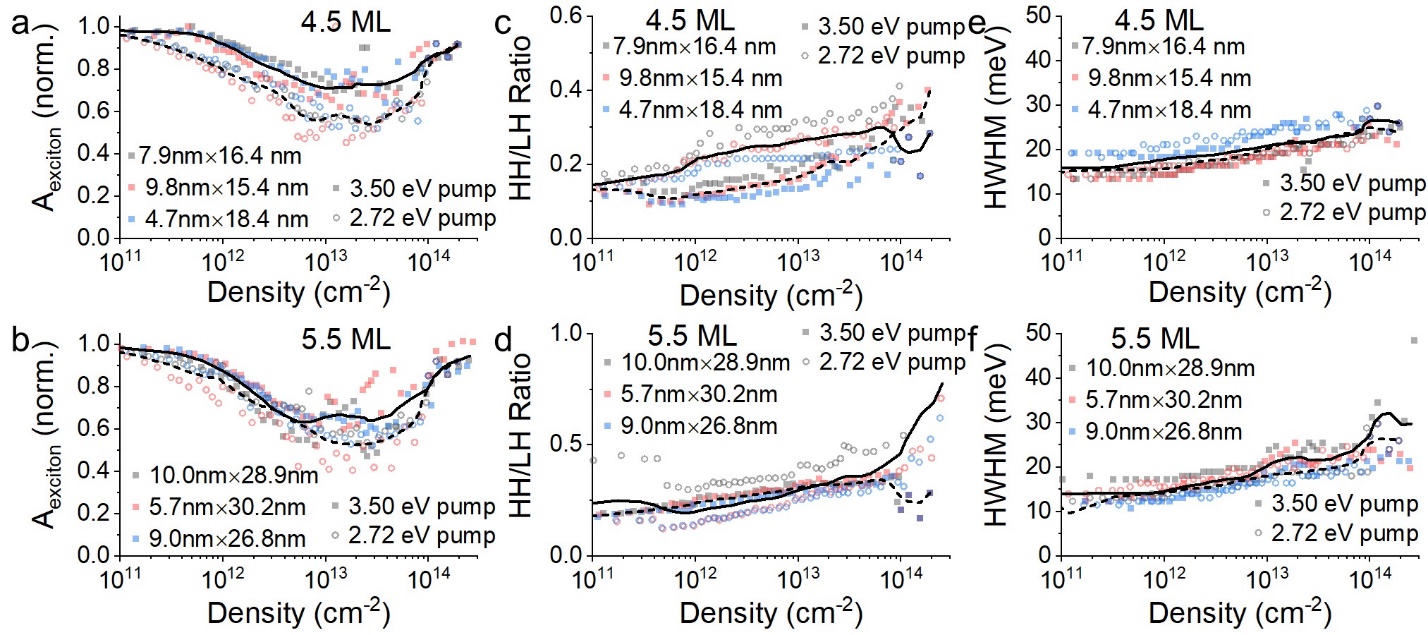


Figure S9. (a, b) Normalized exciton absorption intensity at a pump-probe delay of 40 ps for three (a) 4.5 and (b) 5.5 ML CQW samples as a function of photogenerated sheet density. Each spectrum is collected at a 40 ps pump-probe delay for many powers of 3.50 eV pump light. (c, d) Ratio of heavy hole and light hole bleach signals as a function of photogenerated sheet densities for the same (c) 4.5 and (d) 5.5 ML samples. (e, f) Half-width at half-maximum (HWHM) of the HH bleach feature from the transient absorption spectrum (ΔA) plotted against photogenerated sheet densities of the same (e) 4.5 and (f) 5.5 ML samples. In all cases, data for measurements with 3.50 eV pump are shown in solid symbols and data for 2.72 eV pump measurements are shown in open symbols. A solid line represents a smoothed average of 3.50 eV pump experiments and a dashed line corresponds to the smoothed average of 2.72 eV pump.


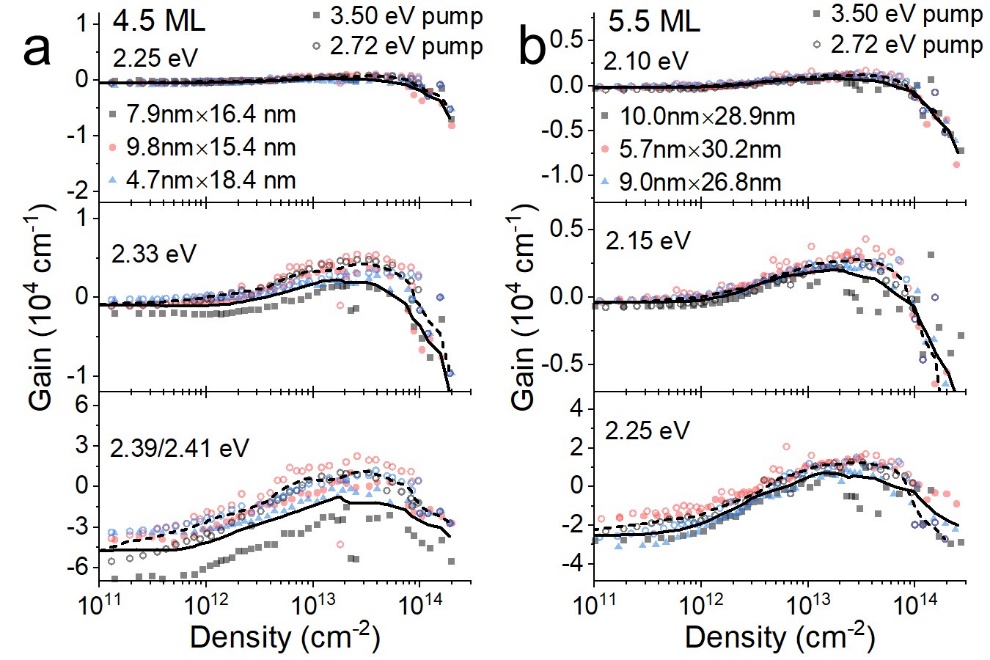


Figure S10. (a, b) Line-cuts of gain or loss at 40 ps pump-probe delay for representative energy values as a function of photogenerated sheet density for (a) 4.5 ML and (b) 5.5 ML CQW samples. In all cases, data for measurements with 3.50 eV pump are shown in solid symbols and data for 2.72 eV measurements are shown in open symbols. A solid line represents a smoothed average of 3.50 eV pump experiments and a dashed line corresponds to the smoothed average of 2.72 eV pump.


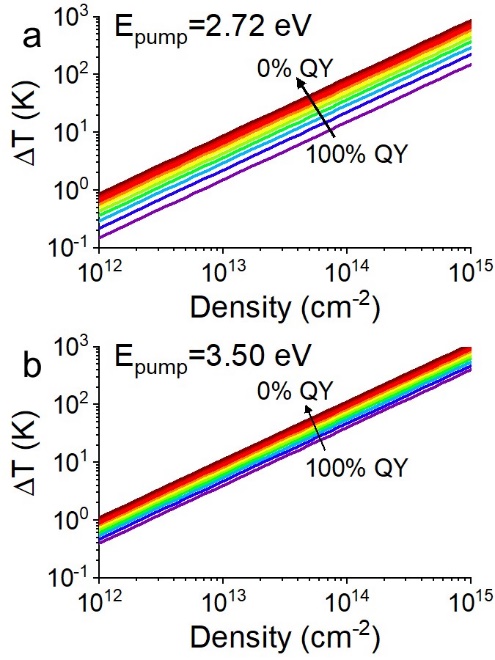


Figure S11. Estimated lattice heating of 5.5 ML CQW under (a) 2.72 eV photon energy excitation and (b) 3.50 eV photon energy excitation, calculated based upon the heat capacity of bulk CdSe at room temperature under different quantum yield assumptions. Absorbed photons are assumed to deposit the entirety of excess energy above the band gap as heat, but additional heating due to non-radiative decay of the band edge excitons is considered based upon the quantum yield.


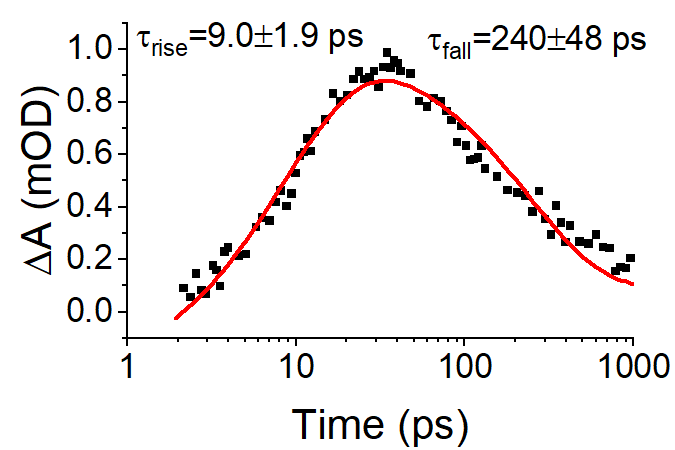


Figure S12. Infrared pump, electronic probe dynamics of 9.0 nm × 26.8 nm 5.5 ML CQWs photoexcited at 0.35 eV on resonance with organic ligand carbon-hydrogen vibrations and probed at 2.22 eV. Rising dynamics reflect heat inflow from the ligands; falling dynamics reflect heat outflow from the heated CQW and ligand complex to the solvent environment.^4,5^


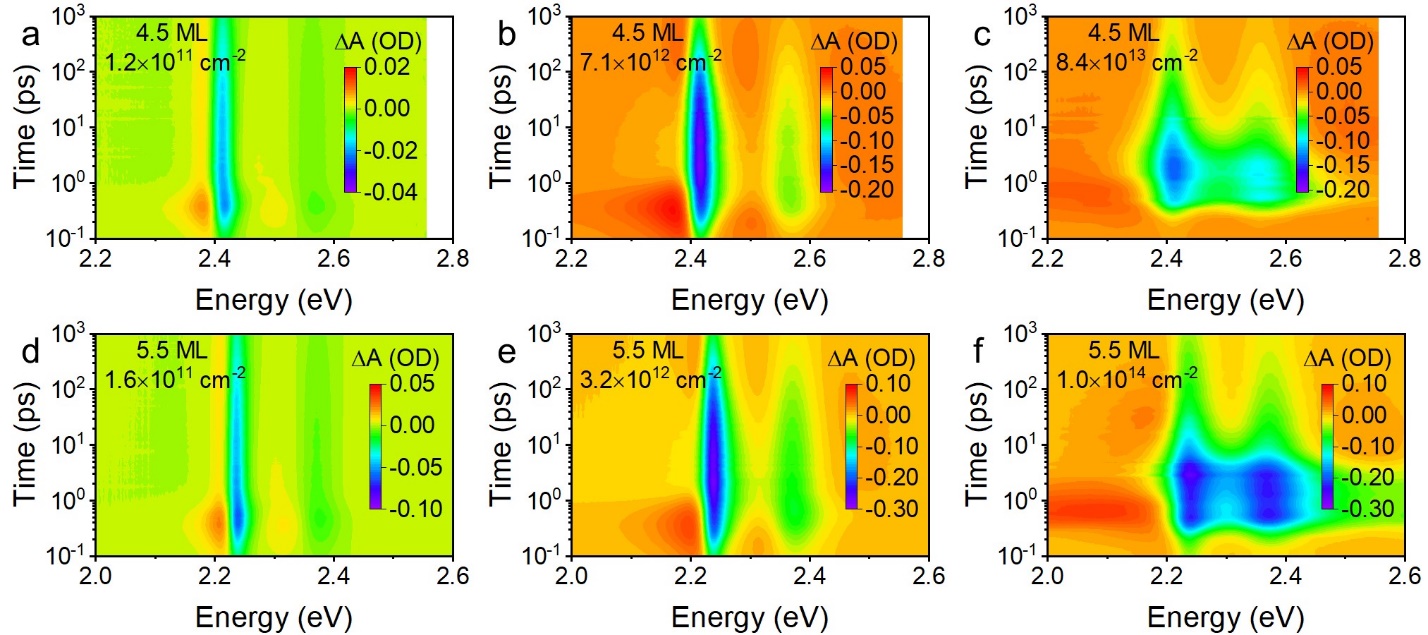


Figure S13. (a-f) Two-dimensional maps of time- and energy-resolved transient absorption (ΔA) for select photogenerated sheet densities of (a-c) 4.5 and (d-f) 5.5 ML CQWs. Note that scales of (b) and (c) and (e) and (f) are the same.


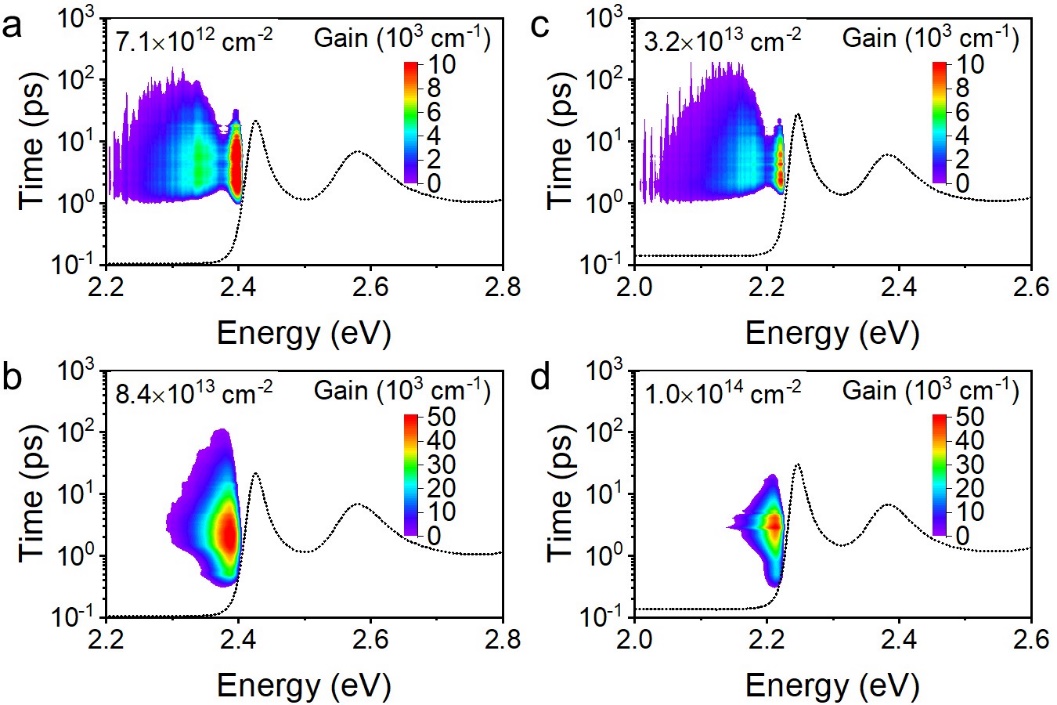


Figure S14. (a, b) Two-dimensional maps of time- and energy-resolved gain of 4.5 ML CQWs at (a) 7.2×10^12^ cm^-2^ and (b) 8.4×10^13^ cm^-2^. (a, b) Two-dimensional maps of time- and energy-resolved gain of 4.5 ML CQWs at (a) 3.2×10^12^ cm^-2^ and (b) 1.0×10^14^ cm^-2^. Regions of loss are shown in white. The dashed overlaid lines are the absorption spectra of the sample. In all cases, data was acquired with a 3.50 eV pump photon energy.


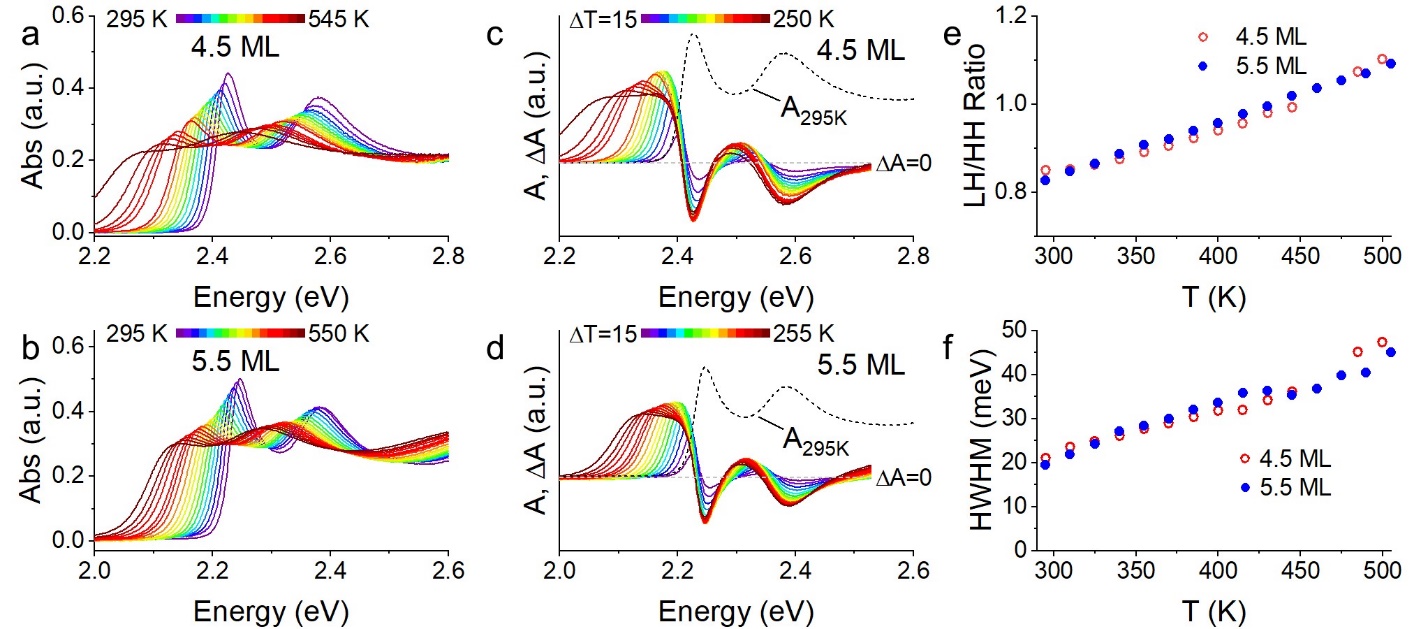


Figure S15. (a, b) Static absorption spectra of (a) 4.5 ML and (b) 5.5 ML CdSe CQWs. (c, d) Corresponding differential absorption spectra in colors, referenced to the absorption at 295 K, shown in the black dashed line. (e) Ratio of the light-hole absorption feature to the heavy-hole (band edge) absorption feature of the static absorption spectra in (a) and (b). (f) Half-width at half-maximum of the band edge absorption feature from static absorption spectra in (a) and (b).


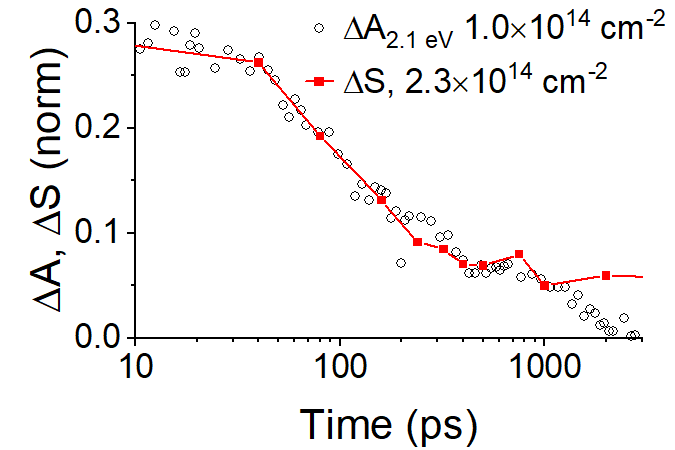


Figure S16. Transient absorption signal (ΔA, open circles) at 2.1 eV of 5.5 ML CQWs excited with 350 nm pump energy to create a sheet density of 1.0×10^14^ cm^-2^ compared to the transient X-ray diffraction scattering signal (ΔS, red squares and line) of a 5.5 ML CQW sample photoexcited with 400 nm pump energy to a density of 2.3×10^14^ cm^-2^.

References

(1) She, C.; Fedin, I.; Dolzhnikov, D. S.; Dahlberg, P. D.; Engel, G. S.; Schaller, R. D.; Talapin, D. V. Red, Yellow, Green, and Blue Amplified Spontaneous Emission and Lasing Using Colloidal CdSe Nanoplatelets. *ACS Nano* **2015**, *9* (10), 9475–9485. https://doi.org/10.1021/acsnano.5b02509.

(2) Ithurria, S.; Talapin, D. V. Colloidal Atomic Layer Deposition (c-ALD) Using Self-Limiting Reactions at Nanocrystal Surface Coupled to Phase Transfer between Polar and Nonpolar Media. *J. Am. Chem. Soc.* **2012**, *134* (45), 18585–18590. https://doi.org/10.1021/ja308088d.

(3) Jasieniak, J.; Smith, L.; Van Embden, J.; Mulvaney, P.; Califano, M. Re-Examination of the Size-Dependent Absorption Properties of CdSe Quantum Dots. *J. Phys. Chem. C* **2009**, *113* (45), 19468–19474. https://doi.org/10.1021/jp906827m.

(4) Diroll, B. T.; Schaller, R. D. Heating and Cooling of Ligand-Coated Colloidal Nanocrystals in Solid Films and Solvent Matrices. *Nanoscale* **2019**, *11* (17), 8204–8209. https://doi.org/10.1039/C9NR01473J.

(5) Diroll, B. T.; Guo, P.; Schaller, R. D. Heat Transfer at Hybrid Interfaces: Interfacial Ligand-to-Nanocrystal Heating Monitored with Infrared Pump, Electronic Probe Spectroscopy. *Nano Lett.* **2018**, *18* (12), 7863–7869. https://doi.org/10.1021/acs.nanolett.8b03640.
